# Molecular diagnosis of an immature *Ascaris lumbricoides* infection with negative parasitological examinations

**DOI:** 10.1016/j.idcr.2026.e02707

**Published:** 2026-07-26

**Authors:** Maryam Ghavami, Eshrat Beigom Kia, Enayat Darabi, Salman Zafari, Zohreh Fakhrieh-Kashan

**Affiliations:** a Department of Medical Parasitology and Mycology, School of Public Health, Tehran University of Medical Sciences, Tehran, Iran; b Student's Scientific Research Center, Tehran University of Medical Sciences, Tehran, Iran

**Keywords:** *Ascaris lumbricoides*, Ascariasis, Parasitological, Immature helminth, Tehran, Iran

## Abstract

Ascariasis is one of the most prevalent parasitic infections worldwide, particularly in developing countries, and is caused by *Ascaris lumbricoides*. Diagnosis can be challenging during the early stages of infection, especially when adult helminths have not yet become established in the intestine and routine parasitological examinations yield negative results. A 40-year-old female patient was referred to a hospital in South Tehran, Iran, with various clinical manifestations, including gastrointestinal symptoms, abdominal cramps, respiratory distress, cough, wheezing, localized skin itching, and blood-tinged sputum. Routine laboratory investigations were within normal limits. On November 28, 2024, following the expulsion of an immature helminth, the specimen was sent to the Helminthology Laboratory, School of Public Health, Tehran University of Medical Sciences, for detailed parasitological examination. Three stool samples were examined using direct smear, formalin–ethyl acetate concentration, zinc sulfate flotation, and agar plate culture techniques. No helminth eggs were detected in any of the samples, and all parasitological examinations yielded negative results. Molecular characterization of the immature helminth was performed using conventional polymerase chain reaction (PCR) targeting the *Cox1* gene, which yielded the expected 520-bp amplicon and confirmed the diagnosis of *A. lumbricoides*. This case underscores the importance of a comprehensive laboratory workup for the diagnosis of ascariasis in patients with heterogeneous clinical presentations, particularly during the early stages of infection. The combined use of molecular and parasitological methods can facilitate the early detection of cases in which adult helminths have not yet developed and routine stool examinations are negative.

## Introduction

Ascariasis, one of the most common helminthic infections caused by *Ascaris lumbricoides*, remains a major public health challenge in developing countries [1]. This nematode is known as a soil-transmitted helminth (STH) that affects humans. Fertilized eggs are produced by the female’s helminth in the intestine and excreted in the feces. The eggs reach the infective stage in about 2–3 weeks in soil and humans can become infected by ingesting contaminated water, food, and vegetables. After the eggs enter the body, the larvae are released in the small intestine and pass through the intestinal wall, enter the bloodstream and travel to the liver and then to the heart and lungs. In the lungs, the larvae remain for about a week, then migrate to the throat and re-enter the digestive tract. The larvae eventually develop into adults in the small intestine [2].

Ascariasis remains a concern, affecting 772–892 million people worldwide, many of whom are asymptomatic and particularly affecting preschool children. The disease is endemic in tropical and subtropical areas of Asia, sub-Saharan Africa, and the Americas, particularly in developing countries [3]. Intestinal obstruction occurs with severe infestation, and if the intestine is perforated, granuloma formation and chronic peritonitis may be observed. In addition, adult helminths can obstruct the bile ducts or pancreas, causing cholangitis, cholecystitis, pancreatitis, or liver abscesses. Other symptoms such as anorexia, nausea, vomiting, diarrhea, bloating, abdominal distension, and discomfort are equally common [4].

Diagnosis is possible by identifying eggs and adult forms of the parasite in feces and the migration of larvae in other body fluids, such as sputum [5]. There may be a patchy infiltrate (Loeffler syndrome) and/or a picture of rope shadows or railroad tracks on the abdomen on chest radiography (X-ray). Eosinophilia, on peripheral blood analysis, is due to larval migration. Immature *A. lumbricoides* infections, which are still in the pre-patent phase before egg production begins, cannot be diagnosed reliably with parasitological methods alone. In contrast, molecular approaches like qPCR are capable of detecting helminth DNA in parasitologically negative samples [6]. Serological methods such as ELISA and Western blot have been developed for the diagnosis of infections caused by STHs, including *A. lumbricoides*. However, these methods have not yet been widely validated and integrated into routine diagnostic programs [7]. The diagnosis of immature *A. lumbricoides* infections remains challenging because conventional parasitological methods primarily rely on the detection of eggs in stool specimens, which may be absent during the early stages of infection or in cases involving immature helminths. As a result, the sensitivity of routine parasitological examinations may be significantly reduced in such cases. Therefore, the present study describes a rare case of immature *A*. *lumbricoides* infection in a woman from Tehran, Iran, who was not diagnosed with ascariasis despite presenting with diverse clinical symptoms and later submitted an immature *A*. *lumbricoides* specimen to the laboratory. Parasitological and molecular examination of the discarded specimen was negative for eggs or other diagnostic stages, and the immature helminth was identified through parasitological methods and *A. lumbricoides* was described using molecular techniques.

## Case presentation

A 40-year-old female patient was referred to one of the hospitals in South Tehran, Iran, with a helminth observation in her stool and clinical symptoms including gastrointestinal symptoms, e.g. abdominal pain, nausea, vomiting, and cramps, shortness of breath, cough, wheezing, weakness and fatigue, localized skin itching, and a feeling of bloody sputum in the mouth. On November 28, 2024, following the expulsion of an immature helminth, the specimen was sent to the Helminthology Laboratory, School of Public Health, Tehran University of Medical Sciences, for detailed parasitological examination. The patient had a history of taking mebendazole, metronidazole, and ciprofloxacin as prescribed by a physician, as well as a history of consuming local vegetables and handling garden soil without gloves.

**Fig. 1:**
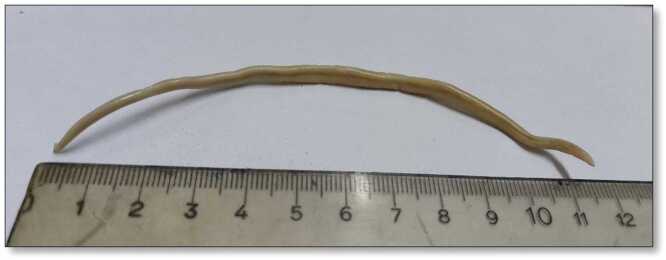
*Ascaris lumbricoides* immature form excreted in the patient's feces.

The helminth recovered from the patient's feces was elongated, cylindrical, with both ends gradually tapering. The specimen was cream to whitish and measured approximately 12.5 cm in length and 2–4 mm in diameter. The anterior extremity was narrower than the posterior end and featured a small, terminal, triradiate oral opening surrounded by three well-developed lips. One lip was positioned dorsally and appeared broadly oval, whereas the remaining two lips were located ventrally. The posterior extremity was conical and straight. Based on these morphological characteristics, the specimen was examined and identified as an immature *A. lumbricoides* at the Helminthology Laboratory, School of Public Health, Tehran University of Medical Sciences (Fig. 1). Furthermore, the relatively small body size of the helminth, together with the absence of eggs in the uterus following puncture of the helminth’s body, provided further evidence supporting its classification as an immature female specimen.

The patient’s laboratory tests, including complete blood count (CBC) (Table 1), liver and kidney function enzymes, and electrolytes, were all within normal limits (Table 2). Ultrasound of the abdomen, pelvis, and appendix did not reveal any abnormalities (obstruction, mass lesion, inflammation, or evidence of helminths). Colonoscopy was also normal and no evidence of bleeding, inflammation, polypoid lesions, or direct presence of helminths in the colon was seen.

**Table 1:** Complete blood count (CBC) of the patient.

**Test**	**Result**	**Unit**	**Reference Value**
WBC	9.9	× 10^3^/ µl	4–10
RBC	4.25	× 10^6^/ µl	3.9–5.8
Hb	12	g/dl	12–17
Hct	37.7	%	36–53
M.C.V	88.7	fl	Adult: 80–100
M.C.H	28.2	p.g	27–32
M.C.H.C	31.8	gr/dl	31–36
Platelets	185,000	µl	150,000–450,000
Lymphocytes	26.1	%	Adult: 25–45
Neutrophils	65.5	%	47–76
RDW-CV	13	%	> 10 years: 11.5–14.5

**Table 2:** Biochemical analysis of the patient's blood.

**Test**	**Result**	**Unit**	**Reference Value**
Urea	36	Mg/dl	Female (20–50 y) = 15–40
Creatinine	0.9	Mg/dl	Female: 0.6–1.3
Amylase	40	U/L	Up to 100
ALP	197	U/L	Female: 64–306
AST	21	IU/L	Female: < 31
ALT	8	U/L	Female: < 32
LDH	340	U/I	Adult: (up to 65 y) < 480
Blood Sugar	100	Mg/dl	< 140
Sodium	134	mEq/L	135–145
Potassium	3.7	mEq/L	3.5–5.5

**Fig. 2:**
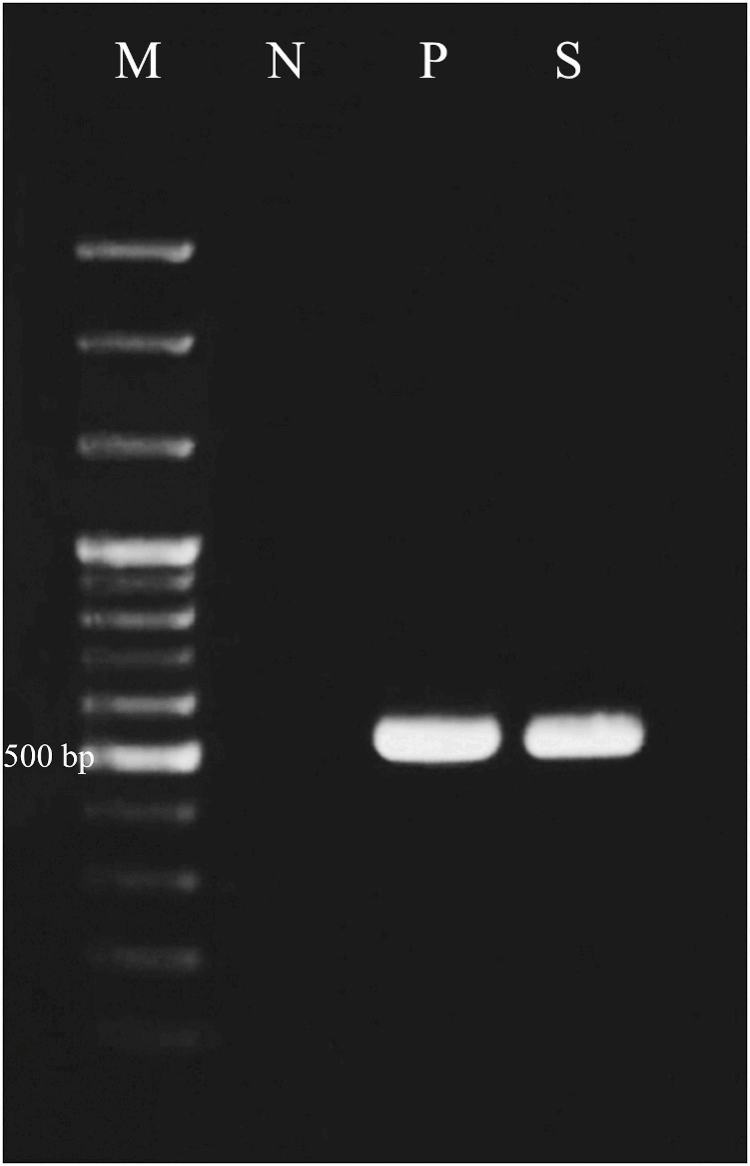
PCR amplification of *Ascaris lumbricoides Cox*1 gene from a woman. M: DNA marker, N: negative control, P: positive control, S: specimen.

**Fig. 3:**
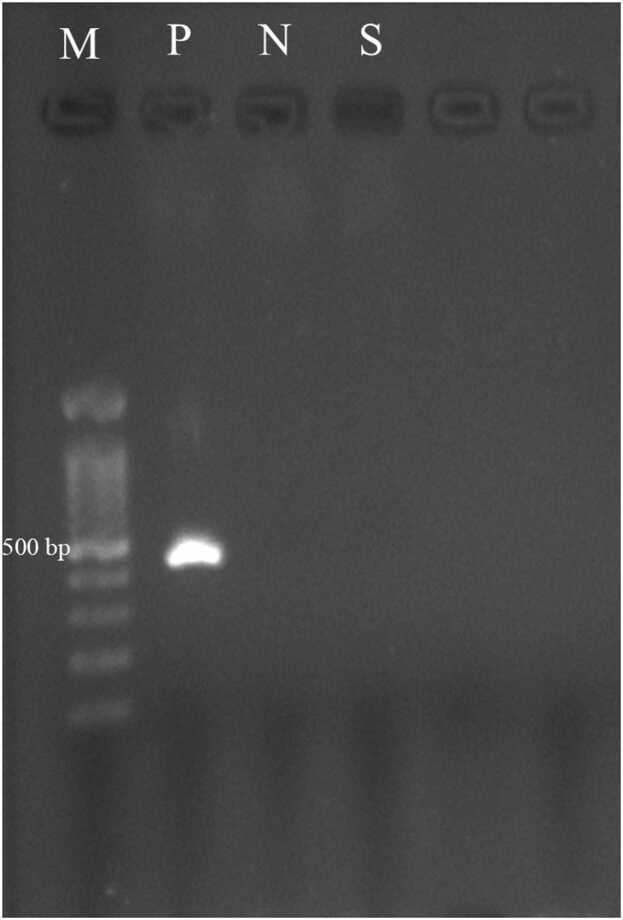
PCR amplification of stool sample of patient, *Cox*1 gene. M: DNA marker, P: positive control, N: negative control, S: specimen.

Stool samples were collected three times at 24-hour intervals, and parasitological tests such as direct smear, formalin-ethyl acetate concentration, zinc sulfate precipitation method, and agar plate culture to investigate infection with *A. lumbricoides* and other intestinal nematodes were negative. The helminth excreted from the patient was initially identified as *A. lumbricoides* based on morphological characteristics; however, it was considered an immature form due to its small size and the absence of eggs in parasitological examinations. Molecular PCR analysis generated a 520 bp amplicon, which was confirmed by sequencing to be *A. lumbricoides* (Fig. 2). However, PCR analysis of the stool sample containing immature helminths yielded no amplification product (Fig. 3).

Genomic DNA was extracted from the helminth specimen patient's stool stored in 70% ethanol using the SinaPure DNA kit (CinaClon, Tehran, Iran) according to the manufacturer's instructions. Polymerase chain reaction (PCR) was applied to amplify cytochrome-c oxidase subunit1 (*Cox*1) mitochondrial gene using F (5′-ATTATTACTGCTCATGCTATTTTGATG-3′) and R (5′-CAAAACAAATGTTGATAAATCAAAGG-3′) primers that amplify 520 bp products [8]. DNA of *A. lumbricoides* available in the Department of Medical Parasitology, School of Public Health, Tehran University of Medical Sciences was used as positive control, and distilled water as negative control. PCR reaction mixtures (25 µl of total volume) included PCR buffer 1 × (10 mM Tris–HCl, pH 8.8, and 50 mM KCl), 1.5 mM MgCl2, 2.5 U/µl of Taq DNA polymerase, 1.25 µM of each dNTPs, 20 pmol each primer, and 5 µl of the DNA sample. The PCR condition consisted of one cycle of initial denaturation at 95 °C for 15 min, followed by 35 cycles including denaturation at 95 °C for 30 s, annealing at 55 °C for 40 s, extending at 72 °C for 1 min, and an additional cycle with a 10 min chain elongation at 72 °C. The PCR products were electrophoresed in 2% agarose gels and Tris–borate–EDTA buffer. It should be noted that, in cases where patients present with clinical symptoms of ascariasis but the helminths have not yet matured and no eggs are detected in parasitological examinations, the use of molecular techniques on stool samples prior to any anti-parasitic treatment can facilitate timely and accurate diagnosis. This approach may help prevent complications such as larval migration within the host, reduce the risk of misdiagnosis, and minimize patient distress.

## Discussion

This study investigated *A. lumbricoides* infection in a 40-year-old female patient using parasitological and molecular methods. The consumption of unwashed or inadequately sanitized vegetables can contribute to *A. lumbricoides* infections in peri-urban areas of large cities. Vegetables grown in or near urban outskirts, often irrigated with contaminated water or fertilized with untreated human waste, can carry eggs, which remain viable in soil due to their resilience [9]. In densely populated peri-urban settings, where access to proper sanitation and clean water may be limited, consuming these vegetables without thorough washing increases the risk of ingesting *Ascaris* eggs, leading to infection [10]. Effective vegetable sanitization and improved sanitation systems are critical to reducing *Ascaris* infections in such regions.

Parasitological tests have low sensitivity, cannot be generalized to different settings, and are influenced by the severity of the infection [11]. In the present study, parasitological examination of the stool sample did not reveal any *A. lumbricoides* eggs. The absence of eggs is likely explained by the immature developmental stage of the helminth, which was present in the host before and after its expulsion and had not yet attained reproductive maturity. But other studies suggest that the presence of *A. lumbricoides* eggs in a patient's feces can be reported when adult helminths are present in the intestine [12], [13]. The helminth isolated in our study was initially diagnosed as *A. lumbricoides* based on morphological features; however, due to its smaller size and the absence of eggs, it was identified as an immature form.

Conventional parasitological methods, including microscopic examination of eggs, larvae, and adult helminths, despite their widespread use in the diagnosis of helminths, have limitations for accurate taxonomic identification [14]. The high similarity of morphological features between closely related species, especially in eggs and larvae, means that in many cases identification is only possible to the genus or even the general group level. In addition, poor specimen quality, inadequate fixation or clarification, and the use of immature stages can obscure diagnostic structures and reduce the accuracy of identification [15]. On the other hand, in mixed infections or in cases where isomorphic species are present, reliance solely on morphological data can lead to misidentification and errors in epidemiological and taxonomic interpretations [16]. Therefore, in many helminths, conventional methods must be supported by complementary tools such as molecular studies and “integrative taxonomy” approaches.

Integrative taxonomy, described in 2005, is an approach that does not rely solely on morphology to identify and delimit species, but rather combines morphological data with molecular, biological, ecological, and geographic evidence [17], [18]. In helminths, this approach is valuable in examining closely related species that are morphologically similar or have immature developmental stages, as classical methods alone may lead to misidentification [19]. Therefore, integrative taxonomy can increase the accuracy of species identification and provide a stronger basis for studies of epidemiology, biodiversity, and parasite transmission cycles.

Molecular methods, as complementary diagnostic tools, play an important role in the accurate identification of parasitic helminths. These methods are very valuable for epidemiological studies, transmission tracing, and genetic diversity assessment. Therefore, various PCR methods and sequencing of target genes such as 28S rDNA or ITS regions are often used alongside classical methods to make taxonomic identification more reliable [20]. The result of the molecular test of this study showed an amplification of the *Cox*1 gene of *A. lumbricoides* at Ca. 520 bp. Molecular tests are not usually used in the diagnosis of large helminths that can be distinguished by morphological methods, but they have been used in cases such as the immature helminth, differentiation of similar species, e.g. *A. lumbricoides* from *A. suum* [21] or their genotyping [7]. It should be noted that the combined use of parasitological and molecular methods is particularly useful in cases of low parasite burden or immature helminths, improving diagnostic accuracy, reducing the risk of clinical misinterpretation, and facilitating timely treatment. Although molecular approaches enhance both research and clinical outcomes, limitations such as high cost, the need for specialized equipment, and the requirement for high-quality DNA remain significant challenges.

Clinicians should consider molecular testing as a complementary tool when morphological diagnosis is inconclusive for various reasons, specimen quality is inadequate, or differentiation between closely related and isomorphic species is of particular importance [22]. In the case of ascariasis, although stool examination is the primary diagnostic method, PCR and targeted gene sequencing can complement the diagnosis in doubtful or specific cases such as pregnancy, mixed infections, or the need for precise taxonomic confirmation.

## Conclusion

A combined diagnostic approach incorporating parasitological and molecular methods for the detection of *A. lumbricoides* infection in patients with heterogeneous clinical manifestations, particularly during the early stages of infection when adult helminths have not yet developed and routine stool examinations are often negative, can facilitate the timely diagnosis of this parasite. Such an integrated diagnostic strategy can improve diagnostic accuracy and reduce the risk of missed or delayed diagnosis during the early phase of infection. In addition, the application of molecular techniques alongside conventional diagnostic methods may enhance the detection of atypical or low-intensity infections, thereby supporting more effective clinical management and disease control. Furthermore, future studies involving a larger number of *A. lumbricoides* isolates from Iran and other regions of the world could provide deeper insights into the molecular characteristics, phylogenetic relationships, genetic diversity, population structure, and evolutionary patterns of this parasite, thereby contributing to a better understanding of its epidemiology, transmission dynamics, and the development of more effective control strategies.

## CRediT authorship contribution statement

**Maryam Ghavami:** Conceptualization, Investigation, Methodology, Writing – original draft. **Eshrat Beigom Kia:** Conceptualization, Investigation. **Enayat Darabi:** Investigation, Methodology. **Salman Zafari:** Investigation, Methodology. **Zohreh Fakhrieh-Kashan:** Conceptualization, Supervision, Work administration, Writing − review & editing. All authors reviewed and approved the manuscript.

## Ethics

This study was approved by the Research Ethics Committee of the Tehran University of Medical Sciences, Tehran, Iran (Code No. IR.TUMS.SPH.REC.1404.208). The patient was informed that her personal information would remain confidential and informed written consent was obtained for the publication.

## Funding

This study was conducted with the support of the Vice Chancellor for Research and Technology, 10.13039/501100004484Tehran University of Medical Sciences, Tehran, Iran (Project no. 91546-125-6-1404).

## Declaration of Competing Interest

No potential conflict of interest was reported by the authors.

## Data Availability

The data supporting the findings of this report are available from the corresponding author upon reasonable request.
